# Electronic Implementation of a Deterministic Small-World Network: Synchronization and Communication

**DOI:** 10.3390/e25050709

**Published:** 2023-04-25

**Authors:** Daniel Reyes-De la Cruz, Rodrigo Méndez-Ramírez, Adrian Arellano-Delgado, César Cruz-Hernández

**Affiliations:** 1 Electronics and Telecommunication Department, Scientific Research and Advanced Studies Center of Ensenada, Ensenada 22860, Mexico; 2 National Council of Science and Technology, Ciudad de Mexico 03940, Mexico; 3 Engineering, Architecture and Design Faculty, Autonomous University of Baja California, Ensenada 22860, Mexico

**Keywords:** deterministic network, small-world network, communication, synchronization, FPGA, Chua’s chaotic circuit

## Abstract

In this paper, synchronization and encrypted communication transmissions of analog and digital messages in a deterministic small-world network (DSWN) are presented. In the first instance, we use a network with 3 coupled nodes in a nearest-neighbor (NN) topology, then the amount of nodes is increased until reaching a DSWN with 24 nodes. The synchronization and encrypted communication transmissions using a DSWN are presented experimentally by using Chua’s chaotic circuit as node, in both analog and digital electronic implementations, where for the continuous version (CV) we use operational amplifiers (OA), and in the discretized version (DV) we use Euler’s numerical algorithm implemented in an embedded system by using an Altera/Intel FPGA and external digital-to-analog converters.

## 1. Introduction

Many real-life phenomena, such as biological networks, electrical networks, social networks, etc., are modeled as complex dynamical networks that follow certain general and robust patterns, for example, a large number of interconnections among the nodes that integrate the networks. In some cases, the nodes work as team to achieve objectives that would be difficult to reach for a single node. The nodes that integrate these types of networks are modeled as dynamic systems by non-linear differential equations, linear piece-wise, or chaotic maps, and the interactions among the nodes present instantaneous behaviors or with time delays. The synchronization state can converge to a periodic or chaotic trajectories which depend on the initial conditions and/or the parameter values, where in some cases multi-stability can occur.

Recent studies have attempted modeling the behaviors of a particular type of networks, referred to as small-world (SW) networks, with *Watts* and *Strogatz* being pioneers in the study of these type of networks [1]. A model of an SW network starts with a NN topology network, and subsequently, based on a probability *p* (represented in the interval 0≤p≤1), connections are added in the original NN network to obtain an SW network. An SW network presents two main characteristics: a high clustering coefficient and a low average path length. The current literature reports many papers related to SW networks, for example, see [2,3,4,5,6,7].

Furthermore, Comellas et al. in [8] present a deterministic SW model to streamline the flow of information in wireless communication networks. *Comellas* and *Sampels* present a deterministic SW network as an alternative to stochastic models in order to calculate relevant parameters of the SW network by using a simplest method [9]. Zhang and Rong [10] present a deterministic model created by edge iterations. In 2010, Zhang et al. published a deterministic model with different weights connections in order to improve the performance in the transmission of packets in communication networks, see [11]. Given the random nature of small-world stochastic networks, they have the disadvantage that the resulting complex network topology is unknown when the number of nodes or connections are varied. For this reason, the DSWN presents an advantageous alternative over the stochastic models, since they allow us a direct calculation of the relevant parameters of the network, for example, average degree, grade distribution, clustering coefficient, average path length, and diameter of the network. On the other hand, the synchronization of complex systems has been studied in regular, irregular, and random networks, see [12,13,14,15]; therefore, this study seeks to achieve synchronization in DSWNs.

This work is organized as follows: In Section 2, a brief review on synchronization of complex networks is presented. Section 3 describes the used algorithm to generate a DSWN. In Section 4, synchronization of a DSWN network using Chua’s chaotic circuit as node is presented. Section 5 describes the experimental synchronization and communication of a DSWN by using six Chua’s circuits as nodes, where the electronic representation in its CV is implemented using operational amplifiers (OA) in order to develop private communications. Section 6 describes the experimental synchronization and communication of a DSWN by using six Chua’s circuits as nodes, where its DV is obtained using Euler’s numerical algorithm, and a digital communication and its implementation are conducted by using an FPGA as the embedded system. Finally, in Section 7 a conclusion is presented.

## 2. Brief Review on Synchronization of Complex Networks

### 2.1. Synchronization of Complex Network

We consider a complex network composed of *N* identical nodes, linearly and diffusively coupled through the first state of each node. In this network, each node constitutes a *n*-dimensional dynamical system, described as follows:

(1) $${\dot{x}}_{i}=f\left(x_{i}\right)+u_{i},\;\;\;\;\;i=1,2,\ldots ,N,$$

where $x_{i}={\left(x_{i1},\;x_{i2},\;\ldots ,\;x_{in}\right)}^{T}\in R^{n}$ are the state variables of the node *i*, $u_{i}={\left(u_{i1},0,\;\ldots ,0\right)}^{T}$$\in R^{n}$ is the input signal of the node *i*, and is defined by

(2) $$u_{i}=c\sum_{j=1}^{N}a_{ij}\Gamma x_{j},\;\;\;\;\;i=1,2,\ldots ,N,$$

the constant c>0 represents the *coupling strength* of the complex network and $\Gamma \in R^{n\times n}$ is a constant 0−1 matrix linking coupled state variables. For simplicity, we assume that $\Gamma =diag\left(r_{1},\;r_{2},\ldots ,r_{n}\right)$ is a diagonal matrix with $r_{i}=1$ for a particular *i* and $r_{j}=0$ for j≠i, this means that two coupled nodes are linked through their i−th state variables, whereas $A=\left(a_{ij}\right)\in R^{N\times N}$ is the *coupling matrix*, which represents the coupling topology of the complex network. If there is a connection between node *i* and node *j*, then $a_{ij}=1$; otherwise $a_{ij}=0$ for i≠j. The diagonal elements of coupling matrix A are defined as

(3) $$a_{ii}=-\sum_{j=1,\;j\neq i}^{N}\;a_{ij}=-\sum_{j=1,\;j\neq i}^{N}\;a_{ji},\;\;\;\;\;i=1,2,\ldots ,N,$$

If the *degree* of node *i* is $d_{i}$, then $d_{i}=-a_{ii},$ i=1,2,…,N.

Now, suppose that the complex network (1) and (2) is connected without isolated clusters. Then, A is a symmetric irreducible matrix. In this case, it can be shown that zero is an eigenvalue of A with multiplicity 1 and all the other eigenvalues of A are strictly negative [16,17].

Synchronization states of nodes in complex systems can be characterized by the non-zero eigenvalues of A. The complex network (1) and (2) is said to achieve (asymptotically) synchronization if [17]

(4) $$x_{1}\left(t\right)=x_{2}\left(t\right)=\;\ldots \;=x_{N}\left(t\right),\;\mathrm{as}\;t\to \infty .$$

The diffusive coupling condition (3) guarantees that the synchronization state is a solution, $s\left(t\right)\in R^{n}$, of an isolated node, that is

(5) $$\dot{s}\left(t\right)=f\left(s(t)\right),$$

where s(t) can be an *equilibrium point*, a *periodic orbit*, or a *chaotic attractor*. Thus, stability of the synchronization state,

(6) $$x_{1}\left(t\right)=x_{2}\left(t\right)=\;\ldots \;=x_{N}\left(t\right)=s\left(t\right),$$

of complex network (1) and (2) is determined by the dynamics of an isolated node, the coupling strength *c*, the inner linking matrix Γ, and the coupling matrix A.

The dynamics of an isolated node are determined by $\bar{d}$, which is a positive constant, such that zero is an exponentially stable point, the *n*-dimensional isolated system is determined by

(7) $$\left\{\begin{array}{c} {\dot{z}}_{1}=f_{1}\left(z\right)-\bar{d}z_{1},\\ {\dot{z}}_{2}=f_{2}\left(z\right),\\ \;\vdots \\ {\dot{z}}_{n}=f_{n}\left(z\right). \end{array}\right.$$

Note that system (7) corresponds to the mathematical model of an *isolated node* with state feedback $-\bar{d}z_{1}$.

### 2.2. Synchronization Conditions

The following theorem gives the conditions to achieve synchronization of the network (1) and (2) as is established in (4).

**Theorem** **1** ([16,17]). *Consider the dynamical network (1) and (2). Let*

(8) $$0=\lambda_{1}>\lambda_{2}\geq \lambda_{3}\geq \cdots \geq \lambda_{N}$$

*be the eigenvalues of a coupling matrix A. Suppose that there exists an n×n, D>0, and two constants $\bar{d}<0$ and τ>0, such that*

(9) $${\left[Df(s(t))+d\Gamma \right]}^{T}D+D\left[Df(s(t))+d\Gamma \right]\leq -\tau I_{n}$$

*for all $d\leq \bar{d}$, where $I_{n}\in R^{n\times n}$ is an unit matrix. If, moreover,*

(10) $$c\lambda_{2}\leq \bar{d},$$

*then the synchronization state (6) of dynamical network (1) and (2) is exponentially stable.*

Since $\lambda_{2}<0$ and $\bar{d}<0$, inequality (10) is equivalent to

(11) $$c\geq \left|\frac{\bar{d}}{\lambda_{2}}\right|.$$

Therefore, the synchronizability of (1) and (2) with respect to a specific coupling topology can be characterized by the second-largest eigenvalue of A.

## 3. Generator Algorithm of DSWN

In this work, we use the algorithm introduced by Zhongzhi Zhang et al. in 2006, see [10]. A network is denoted as N(l) after the evolution of *l* iterations. In this algorithm, the network grows through an iterative procedure. The algorithm is as follows: for l=0 the initial network N(0) is a triangle that contains three coupled nodes in an NN topology, For l=1, the network N(l) is obtained from N(l−1) by adding a new node for each connection created in the step l−1 and connecting it to the nearest nodes. The algorithm can be summarized as follows: in each step iteration, for each edge that exists in the network, a new node is added and connected to its two nearest neighbors, see details in [10].

Furthermore, according to [10], the total number of nodes $N_{T}\left(l\right)$ for each iteration *l* is as follows:

(12) $$N_{T}\left(l\right)=N_{T}\left(0\right)\cdot 2^{l},$$

where $N_{T}\left(0\right)=3$.

With respect to the number of edges $N_{e}\left(l\right)$ added to each iteration, we have the following

(13) $$N_{e}\left(l\right)=N_{e}\left(0\right)\cdot 2^{l+1}-3,$$

where $N_{e}\left(0\right)=3$.

Taking into consideration (12) and (13), the average node degree $\langle k\rangle$ of the network for each iteration *l* is as follows:

(14) $$\langle k\rangle =2\cdot \frac{Ne}{N_{T}}=\frac{2\cdot (N_{e}\left(0\right)\cdot 2^{l+1}-3)}{N_{T}\left(0\right)\cdot 2^{l}}=4\left(1-\frac{1}{2^{l+1}}\right).$$

Generally, SW networks can be identified by three main properties: (*i*) the average path length does not increase logarithmically with the size of the network or with the increase in the number of nodes, but it grows or decreases as the number of nodes varies; (*ii*) the average degree of nodes of the network is small, and (*iii*) the network has a high clustering coefficient.

## 4. Synchronization of a DSWN with Chaotic Chua’s Circuits as Node

### 4.1. Chaotic Chua’s Circuit

In this section, we describe the chaotic Chua’s circuit that we use as node to construct the DSWNs, see [18]. The Chua’s circuit consists of four linear elements (a resistor *R*, an inductor *L*, and two capacitors C1 and C2) and a non-linear element, which is described in [19]. In order to simulate the behavior of the Chua’s circuit in a computer, we used the normalized version described below, see details in [20].

(15) $$\left\{\begin{array}{c} {\dot{x}}_{1}=\alpha \left(x_{2}-x_{1}-f\left(x_{1}\right)\right),\\ {\dot{x}}_{2}=x_{1}-x_{2}+x_{3},\\ {\dot{x}}_{3}=-\beta x_{2}, \end{array}\right.$$

**Figure 1:**
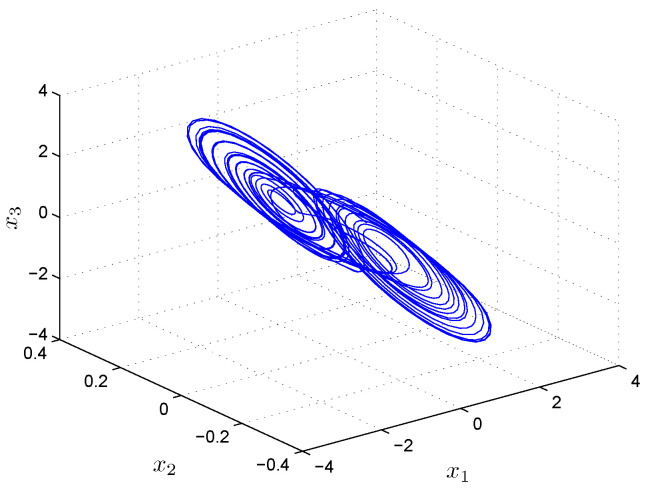
Chaotic attractor generated by the Chua’s circuit (15) and (16).

The non-linearity $f(x_{1})$ is defined as

(16) $$f\left(x_{1}\right)=bx_{1}+\frac{1}{2}\left(a-b\right)\left(\left|x_{1}+1\right|-\left|x_{1}-1\right|\right),$$

where with parameter values α=15.6, β=28, a=−1.143, and b=−0.714, Chua’s circuit generates the chaotic behavior shown in Figure 1.

For $\bar{d}=2.3$ in (7), any isolated chaotic Chua’s circuit (15) and (16) is stabilized, see [16,17]. The state equations for *N* Chua’s circuits in complex dynamical networks according to (1) and (2) can be expressed as follows:

(17) $$\begin{array}{ccc} {\dot{x}}_{i1} & = & \alpha \left(x_{i2}-x_{i1}-f\left(x_{i1}\right)\right)+c\sum_{j=1}^{N}\left(a_{\mathrm{ij}}\Gamma x_{j1}\right),\;i=1,2,\ldots ,N,\\ {\dot{x}}_{i2} & = & x_{i1}-x_{i2}+x_{i3},\\ {\dot{x}}_{i3} & = & -\beta x_{i2}, \end{array}$$

the non-linear functions $f(x_{i1})$, i=1,2,…,N are defined as

(18) $$f\left(x_{i1}\right)=bx_{i1}+\frac{1}{2}\left(a-b\right)\left(\left|x_{i1}+1\right|-\left|x_{i1}-1\right|\right).$$

### 4.2. Synchronization of 24 Chaotic Chua’s Circuits in a DSWN

Now, we present the synchronization of a DSWN for an iteration l=3, which is formed by N=24 chaotic Chua’s circuits. The second eigenvalue $\lambda_{2}=-0.5501$ of the network N(3) is used and the minimum coupling strength (obtained from (11)) to synchronize the network is obtained as follows:

(19) $$\begin{array}{c} c\geq \frac{\left|2.3\right|}{\left|-0.5501\right|}. \end{array}$$

#### Numerical Simulations of DSWN with 24 Chaotic Chua’s Circuits

For the numerical simulations with N=24 chaotic Chua’s circuits, the values of initial conditions in the numerical simulations are chosen as follows:

(20) $$\begin{array}{c} 0\leq x_{i1}\left(0\right)\leq 1,\\ 0\leq x_{i2}\left(0\right)\leq 1,\;\;\;\;\;i=1,2,\ldots ,24.\\ 0\leq x_{i3}\left(0\right)\leq 1, \end{array}$$

**Figure 2:**
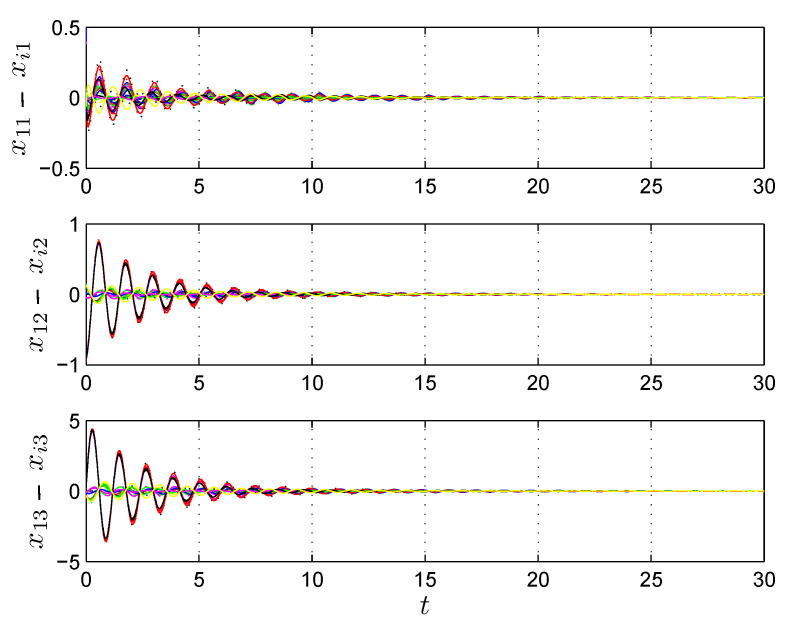
Synchronization error dynamics $x_{11}-x_{i1}$, $x_{12}-x_{i2}$, $x_{13}-x_{i3}$, i=1,2,…,24 of the chaotic Chua’s circuits with c=30, different colors are used for the sole purpose of differentiating the error synchronization signals.

Figure 2 shows the synchronization error dynamics where we can see that the synchronization state for all nodes in the DSWN is convergent.

## 5. Analog Synchronization of Six Chua’s Circuits in a DSWN

This section presents an experimental implementation by using OA and analog components for a potential application with electrical circuits in private communications, by way of illustration we implemented a DSWN for l=1 conformed by the following:

(21) $$N1\left\{\begin{array}{c} {\dot{x}}_{11}=\alpha \left(x_{12}-x_{11}-f\left(x_{11}\right)\right)+u_{1},\\ {\dot{x}}_{12}=x_{11}-x_{12}+x_{13},\\ {\dot{x}}_{13}=-\beta x_{12}, \end{array}\right.$$

(22) $$f\left(x_{11}\right)=bx_{11}+\frac{1}{2}\left(a-b\right)\left(\left|x_{11}+1\right|-\left|x_{11}-1\right|\right)$$

(23) $$N2\left\{\begin{array}{c} {\dot{x}}_{21}=\alpha \left(x_{22}-x_{21}-f\left(x_{21}\right)\right)+u_{2},\\ {\dot{x}}_{22}=x_{21}-x_{22}+x_{23},\\ {\dot{x}}_{23}=-\beta x_{22}, \end{array}\right.$$

(24) $$f\left(x_{21}\right)=bx_{21}+\frac{1}{2}\left(a-b\right)\left(\left|x_{21}+1\right|-\left|x_{21}-1\right|\right)$$

(25) $$N3\left\{\begin{array}{c} {\dot{x}}_{31}=\alpha \left(x_{32}-x_{31}-f\left(x_{31}\right)\right)+u_{3},\\ {\dot{x}}_{32}=x_{31}-x_{32}+x_{33},\\ {\dot{x}}_{33}=-\beta x_{32}, \end{array}\right.$$

(26) $$f\left(x_{31}\right)=bx_{31}+\frac{1}{2}\left(a-b\right)\left(\left|x_{31}+1\right|-\left|x_{31}-1\right|\right)$$

(27) $$N4\left\{\begin{array}{c} {\dot{x}}_{41}=\alpha \left(x_{42}-x_{41}-f\left(x_{41}\right)\right)+u_{4},\\ {\dot{x}}_{42}=x_{41}-x_{42}+x_{43},\\ {\dot{x}}_{43}=-\beta x_{42}, \end{array}\right.$$

(28) $$f\left(x_{41}\right)=bx_{41}+\frac{1}{2}\left(a-b\right)\left(\left|x_{41}+1\right|-\left|x_{41}-1\right|\right)$$

(29) $$N5\left\{\begin{array}{c} {\dot{x}}_{51}=\alpha \left(x_{52}-x_{51}-f\left(x_{51}\right)\right)+u_{5},\\ {\dot{x}}_{52}=x_{51}-x_{52}+x_{53},\\ {\dot{x}}_{53}=-\beta x_{52}, \end{array}\right.$$

(30) $$f\left(x_{51}\right)=bx_{51}+\frac{1}{2}\left(a-b\right)\left(\left|x_{51}+1\right|-\left|x_{51}-1\right|\right)$$

(31) $$N6\left\{\begin{array}{c} {\dot{x}}_{61}=\alpha \left(x_{62}-x_{61}-f\left(x_{61}\right)\right)+u_{6},\\ {\dot{x}}_{62}=x_{61}-x_{62}+x_{63},\\ {\dot{x}}_{63}=-\beta x_{62}, \end{array}\right.$$

(32) $$f\left(x_{61}\right)=bx_{61}+\frac{1}{2}\left(a-b\right)\left(\left|x_{61}+1\right|-\left|x_{61}-1\right|\right)$$

where the inputs signals $u_{i}$, i=1,2,⋯,6 are as follows:

(33) $$\begin{array}{c} u_{1}=-4x_{1}+x_{2}+x_{3}+x_{4}+x_{6},\\ u_{2}=x_{1}-4x_{2}+x_{3}+x_{4}+x_{5},\\ u_{3}=x_{1}+x_{2}-4x_{3}+x_{5}+x_{6},\\ u_{4}=x_{1}+x_{2}-2x_{4},\\ u_{5}=x_{2}+x_{3}-2x_{5},\\ u_{6}=x_{1}+x_{3}-2x_{6} \end{array}$$

with parameter values α=15.6, β=28, a=−1.143, and b=−0.714. Figure 3, Figure 4 and Figure 5 show the electronic diagrams of the six Chua’s circuits corresponding to the nodes (21)–(32).

**Figure 6:**
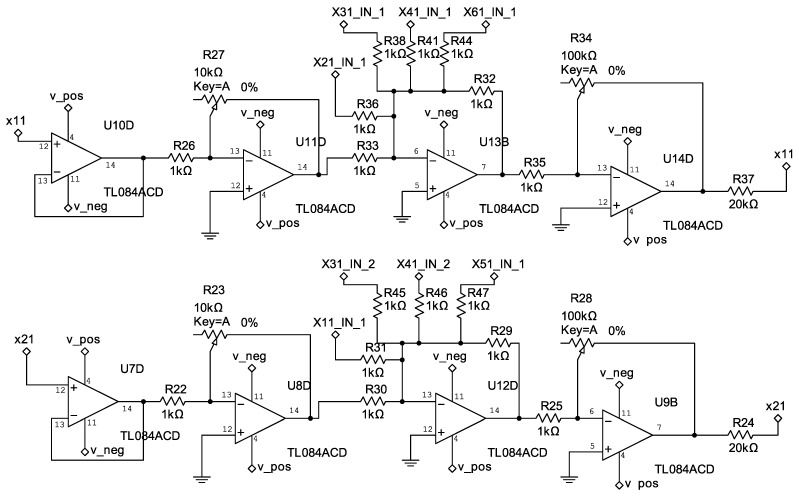
Electrical diagram of the controllers $u_{1}$ and $u_{2}$.

**Figure 7:**
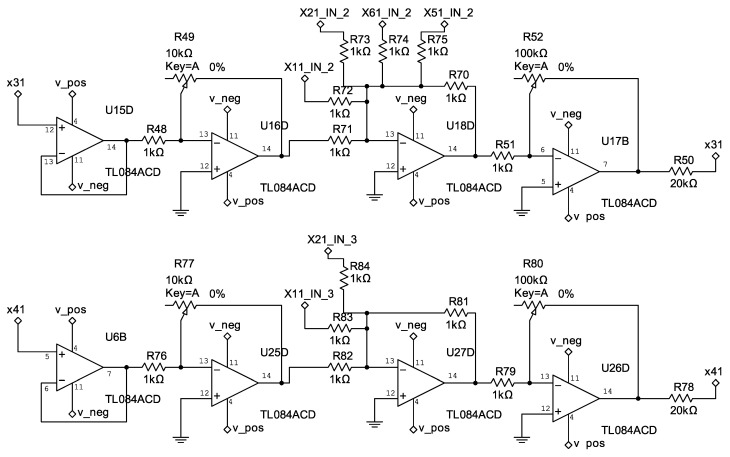
Electrical diagram of the controllers $u_{3}$ and $u_{4}$.

**Figure 8:**
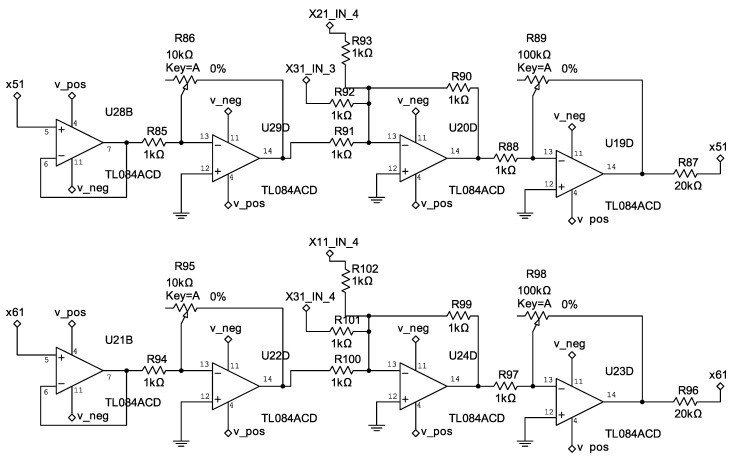
Electrical diagram of the controllers $u_{5}$ and $u_{6}$.

The control circuits corresponding to Equation (33) are shown in Figure 6, Figure 7 and Figure 8.

**Figure 9:**
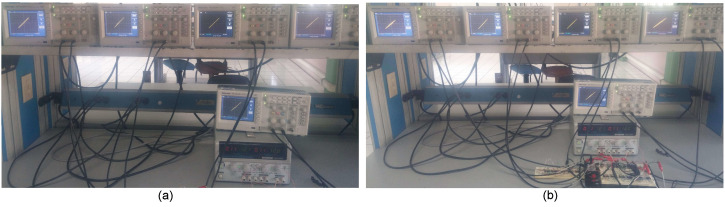
(**a**) Phase portrait of states $x_{11}$ versus $x_{i1}$, (**b**) phase portrait of states $x_{12}$ versus $x_{i2}$ for 6 chaotic Chua’s circuits.

Figure 9a,b show the phase planes of the states $x_{11}$ versus $x_{i1}$ and $x_{12}$ versus $x_{i2}$ of the chaotic Chua’s circuits, with i=1,2,⋯,6, respectively.

### 5.1. Experimental Application for analog Encryption in a DSWN

**Figure 10:**
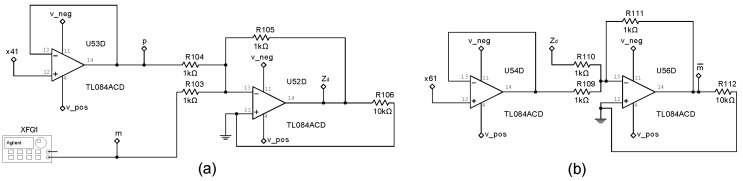
(**a**) Electrical diagram to generate a $Z_{d}$ cryptogram and send it from node Tx (N4), (**b**) electrical diagram to recover the message $\bar{m}$ in the node Rx (N6).

This section presents the chaotic encryption and transmission of information using a DSWN with six Chua’s circuits, the encryption is achieved using the network of N(1) previously synchronized. Once the network is synchronized, encrypted analog messages can be transmitted (Tx) from any node in the network and can be received (Rx) and decrypted in any other node that integrates the network. By way of illustration, the following example is presented: the message m=sin(2πft), with f=1 khz, is encrypted in the node N4 configured as Tx, and it is recovered in the node N6 configured as Rx. The communication scheme consists of adding the chaotic dynamics of the $x_{41}$ state to the message *m*, therefore the resulting cryptogram is $Z_{d}=x_{41}+m$, see Figure 10a, then, the Rx node N6 uses the chaotic dynamics of the $x_{61}$ state to recover the encrypted message, which results in the recovered message $\bar{m}=Z_{d}-x_{61}$, see Figure 10b.

**Figure 11:**
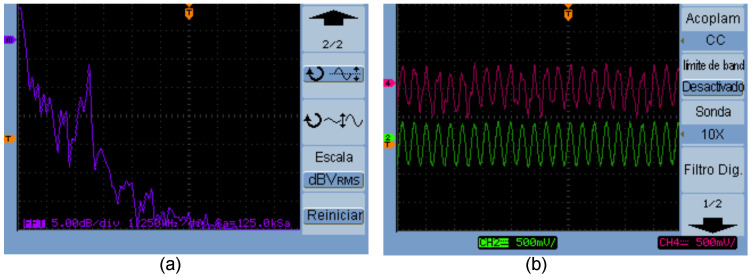
(**a**) Cryptogram in the frequency domain $Z_{d}=x_{41}+m$, (**b**) original message *m* (green line) in N4 and decrypted message $\bar{m}$ (purple line) in node N6.

In Figure 11a, the cryptogram $Z_{d}=x_{41}+m$ is presented in the frequency domain, whereas Figure 11b shows the original message m=sin(2πft) (green line) and the decrypted message $\bar{m}=Z_{d}-x_{61}$ (purple line). From Figure 11a, we can establish that the signal m=sin(2πft) is hidden in the chaotic carrier.

### 5.2. Experimental Application for Bit Encryption in a DSWN

**Figure 12:**
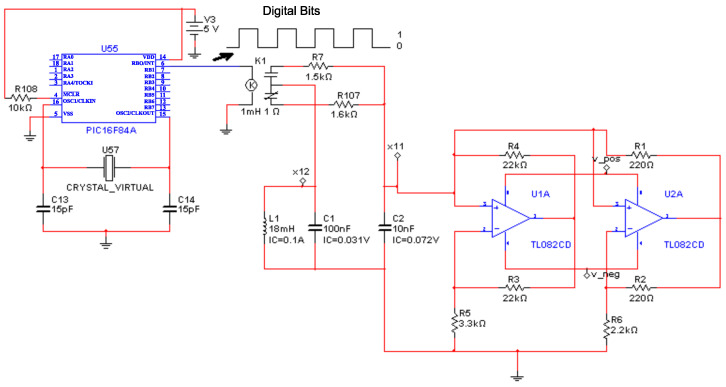
Node N1 as a Tx, N2 to N6 nodes remain unchanged.

In this section, we conducted the encryption of a digitized image using the experimental implementation of the Chua’s circuit. Figure 12 shows the electronic circuit for the encryption process by varying the resistance R7 using a microcontroller microchip P16F84A, where we chose node N1 as a Tx.

**Figure 13:**
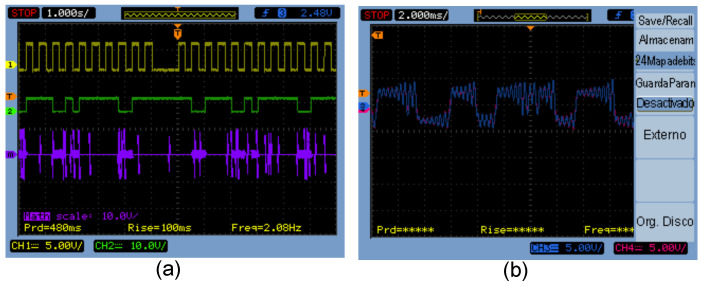
(**a**) Transmission of encrypted digital data using a DSWN: clock (yellow line), data (green line), and synchrony error (purple line); (**b**) chaotic dynamics of the states $x_{11}$ and $x_{51}$ when Tx sends a 1 binary from N1 to N5.

The binary information of the scanned image was taken from [20]. Only unidirectional bits can be sent in this application, this is because the information flows from the Tx node N1 to the Rx nodes $N_{i}$, i=2,…,6, where this is achieved by adding the electronic circuit of Figure 12 in the node that is selected as the Tx; the Rx nodes remain without modifications. Figure 13a shows the message to be sent in green color (3 bytes) and the synchronization error between nodes N1 and N5 in (purple line), where a 1 binary represents synchrony between systems and a 0 binary represents no synchronization. In the synchrony error (purple signal), there are quite a few unwanted peaks due to the use of a mechanical relay. The synchrony error signal can be recovered using filters to clean the undesired peaks and comparators to restore the information to appropriate voltage levels, in this case the main idea is to show the digital encryption in a general way. Figure 13b shows the chaotic dynamics of the states $x_{11}$ and $x_{51}$, it is observed that the dynamics are similar, this is the case when a binary 1 is sent from N1 to N5.

## 6. Digital Synchronization and Communication of a DSWN Implemented in FPGA

For the digital implementation, we used Euler’s method to approximate the ordinary differential equations (ODEs) and to obtain the model proposed in (21)–(33). Euler’s method is used in order to discretize a continuous system that is derived from Taylor’s series, when the quadratic and upper order term are truncated [21,22], i.e., if we have the following equation

(34) $$\dot{x}=f\left(x\right);\;\;x\left(0\right)=x{}_{0},\;\;x\in R^{n},$$

then the DV using Euler’s method is given by

(35) $$x_{\left(k+1\right)}=x_{\left(k\right)}+\tau f\left(x_{\left(k\right)}\right),$$

where τ is the step size and *k* is the iteration number that represents the time in discrete version. Euler’s numerical algorithm (35) was considered to obtain the DV of the proposed DSWN (21)–(33) as follows:

(36) $$ND1\left\{\begin{array}{c} x_{11(k+1)}=x_{11(k)}+\tau (\alpha \left(x_{12(k)}-h\left(x_{11(k)}\right)\right)\\ \;+k(-4x_{11(k)}+x_{21(k)}+x_{31(k)}+x_{41(k)}+x_{61(k)}),\\ x_{12(k+1)}=x_{12(k)}+\tau \left(x_{11(k)}-x_{12(k)}+x_{13(k)}\right),\\ x_{13(k+1)}=x_{13(k)}+\tau \left(-\beta x_{12(k)}\right). \end{array}\right.$$

(37) $$h\left(x_{11(k)}\right)=m_{1}x_{11(k)}+\frac{1}{2}\left(m_{0}-m_{1}\right)\left(\right|x_{11(k)}+1|-|x_{11(k)}-1\left|\right),$$

(38) $$ND2\left\{\begin{array}{c} x_{21(k+1)}=x_{21(k)}+\tau (\alpha \left(x_{22(k)}-h\left(x_{21(k)}\right)\right)\\ \;+k(x_{11(k)}-4x_{21(k)}+x_{31(k)}+x_{41(k)}+x_{51(k)})),\\ x_{22(k+1)}=x_{22(k)}+\tau \left(x_{21(k)}-x_{22(k)}+x_{23(k)}\right),\\ x_{23(k+1)}=x_{23(k)}+\tau \left(-\beta x_{22(k)}\right). \end{array}\right.$$

(39) $$h\left(x_{21(k)}\right)=m_{1}x_{21(k)}+\frac{1}{2}\left(m_{0}-m_{1}\right)\left(\right|x_{21(k)}+1|-|x_{21(k)}-1\left|\right),$$

(40) $$ND3\left\{\begin{array}{c} x_{31(k+1)}=x_{31(k)}+\tau (\alpha \left(x_{32(k)}-h\left(x_{31(k)}\right)\right)\\ \;+k(x_{11(k)}-x_{21(k)}-4x_{31(k)}+x_{51(k)}+x_{61(k)})),\\ x_{32(k+1)}=x_{32(k)}+\tau \left(x_{21(k)}-x_{32(k)}+x_{33(k)}\right),\\ x_{33(k+1)}=x_{33(k)}+\tau \left(-\beta x_{32(k)}\right). \end{array}\right.$$

(41) $$h\left(x_{31(k)}\right)=m_{1}x_{31(k)}+\frac{1}{2}\left(m_{0}-m_{1}\right)\left(\right|x_{31(k)}+1|-|x_{31(k)}-1\left|\right),$$

(42) $$ND4\left\{\begin{array}{c} x_{41(k+1)}=x_{41(k)}+\tau (\alpha \left(x_{42(k)}-h\left(x_{41(k)}\right)\right)\\ \;+k(x_{11(k)}+x_{21(k)}-2x_{41(k)})),\\ x_{42(k+1)}=x_{42(k)}+\tau \left(x_{41(k)}-x_{42(k)}+x_{43(k)}\right),\\ x_{43(k+1)}=x_{43(k)}+\tau \left(-\beta x_{42(k)}\right). \end{array}\right.$$

(43) $$h\left(x_{41(k)}\right)=m_{1}x_{41(k)}+\frac{1}{2}\left(m_{0}-m_{1}\right)\left(\right|x_{41(k)}+1|-|x_{41(k)}-1\left|\right),$$

(44) $$ND5\left\{\begin{array}{c} x_{51(k+1)}=x_{51(k)}+\tau (\alpha \left(x_{52(k)}-h\left(x_{51(k)}\right)\right)\\ \;+k(x_{21(k)}+x_{31(k)}-2x_{51(k)})),\\ x_{52(k+1)}=x_{52(k)}+\tau \left(x_{51(k)}-x_{52(k)}+x_{53(k)}\right),\\ x_{53(k+1)}=x_{53(k)}+\tau \left(-\beta x_{52(k)}\right). \end{array}\right.$$

(45) $$h\left(x_{51(k)}\right)=m_{1}x_{51(k)}+\frac{1}{2}\left(m_{0}-m_{1}\right)\left(\right|x_{51(k)}+1|-|x_{51(k)}-1\left|\right),$$

(46) $$ND6\left\{\begin{array}{c} x_{61(k+1)}=x_{61(k)}+\tau (\alpha \left(x_{62(k)}-h\left(x_{61(k)}\right)\right)\\ +k(x_{11(k)}+x_{31(k)}-2x_{61(k)})),\\ x_{62(k+1)}=x_{62(k)}+\tau \left(x_{51(k)}-x_{62(k)}+x_{63(k)}\right),\\ x_{63(k+1)}=x_{63(k)}+\tau \left(-\beta x_{62(k)}\right). \end{array}\right.$$

(47) $$h\left(x_{61(k)}\right)=m_{1}x_{61(k)}+\frac{1}{2}\left(m_{0}-m_{1}\right)\left(\right|x_{61(k)}+1|-|x_{61(k)}-1\left|\right),$$

(48) $$\left\{\begin{array}{c} x_{1prom(k)}=\frac{1}{6}\left(x_{11(k)}+x_{21(k)}+x_{31(k)}+x_{41(k)}+x_{51(k)}+x_{61(k)}\right),\\ x_{2prom(k)}=\frac{1}{6}\left(x_{12(k)}+x_{22(k)}+x_{32(k)}+x_{42(k)}+x_{52(k)}+x_{62(k)}\right),\\ x_{3prom(k)}=\frac{1}{6}\left(x_{13(k)}+x_{23(k)}+x_{33(k)}+x_{43(k)}+x_{53(k)}+x_{63(k)}\right). \end{array}\right.$$

**Figure 14:**
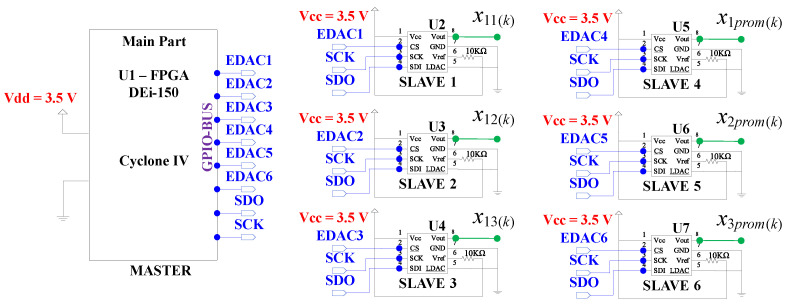
Hardware implementation of the embedded system using the GPIO bus to implement the DSWN (36)–(47).

We used the FPGA Cyclone IV DEi-150 Altera-Intel main-board to design the hardware of the embedded system (ES) to implement the DSWN (36)–(47), which has a general purpose input/output (GPIO) that is configured to connect six external digital-to-analog converters (DACs); all the hardware of the ES is described in Figure 14.

**Figure 15:**
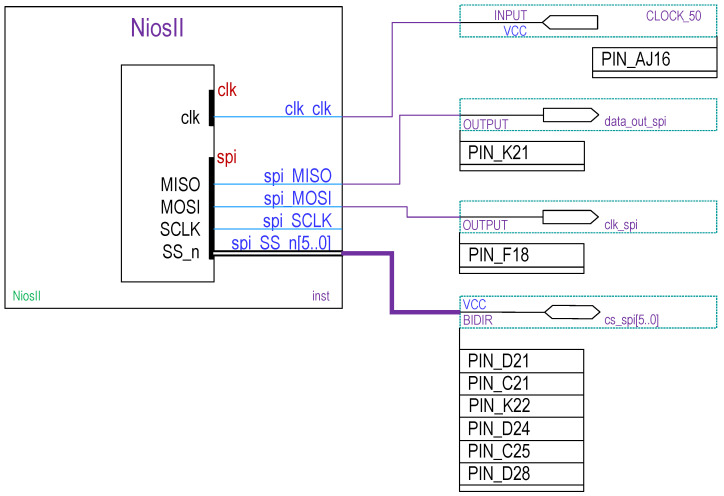
Schematic diagram to implement the processor Nios II fast version within FPGA Cyclone-IV U1 and the pins distribution to set the SPI protocol.

To build the algorithm for the digital circuit implementation of the ES, we used the Quartus II version 12 software, which offers the Qsys tool to design the hardware and software, specifically, the 32-bit embedded main processor Nios II (fast version) and the serial-peripheral-interface (SPI) protocol were implemented inside of the FPGA [23,24]. Subsequently, the SPI port was configured to create the links that use the GPIO port from the FPGA De-i150 main board, which is setting in MOSI (master-output-slave-input) mode where the 32-bit micro-controller in the FPGA generates control signals such as chip-select and clock to set the external DACs, MISO (master-input slave-output) mode is not used. Figure 15 shows the schematic circuit of the FPGA to implement the DV of the DSWN (36)–(47).

The FPGA Cyclone IV DEi-150 was configured in master mode and the GPIO pins were used to reproduce the SPI control signals: SCK, SDO, and SS1-SS6 for each DAC1-DAC6 as slaves, in order to represent the state variables, respectively. The experimental results showed good performance, presenting a time complexity of t=664μs using a clock of 50 MHz. According to the value of the parameter *k*, we proposed to analyze two cases for systems (36)–(47), these are k=0, i.e., the nodes are decoupled, and k=10, i.e., the discretized nodes are coupled.

### 6.1. Uncoupled Nodes

**Figure 16:**
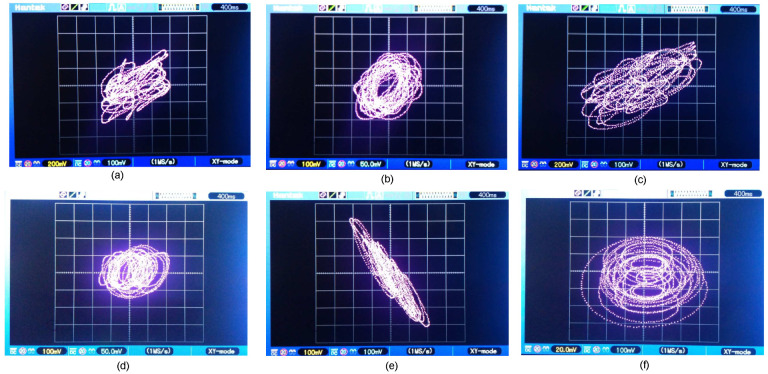
Comparison of the phase planes of the discretized network (36) and (37) and the average states of the network (48) for k=0: (**a**) $x_{11(k)}$ vs. $x_{1prom(k)}$, (**b**) $x_{12(k)}$ vs. $x_{2prom(k)}$, (**c**) $x_{13(k)}$ vs. $x_{3prom(k)}$, (**d**) $x_{1prom(k)}$ vs. $x_{2prom(k)}$, (**e**) $x_{1prom(k)}$ vs. $x_{3prom(k)}$, and (**f**) $x_{2prom(k)}$ vs $x_{3prom(k)}$.

In Figure 16, for k=0, the phase planes of node ND1 are presented corresponding to the discretized network (36) and (37) versus the representation of the average states of the six nodes of the expression (48).

**Figure 17:**
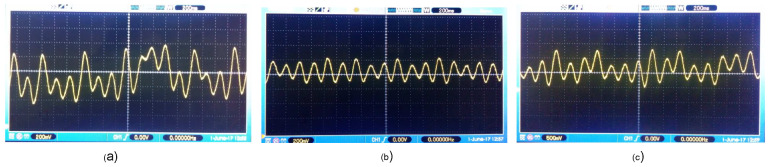
Time evolution of (36) and (37) for k=0: (**a**) $x_{11(k)}$, (**b**) $x_{12(k)}$, and (**c**) $x_{13(k)}$.

Figure 17 shows the time evolution of the node ND1 corresponding to the system (36) and (37).

### 6.2. Coupled Nodes

**Figure 18:**
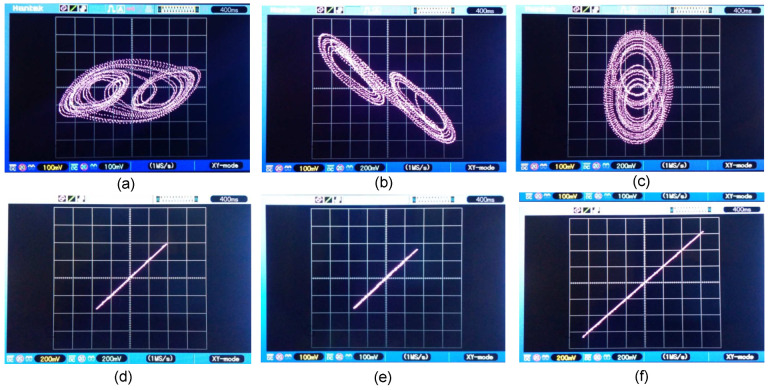
Comparison of the phase planes of system (36) and (37) and the average states of the network (48) using k=10: (**a**) $x_{11(k)}$ vs. $x_{1prom(k)}$, (**b**) $x_{12(k)}$ vs. $x_{2prom(k)}$, (**c**) $x_{13(k)}$ vs. $x_{3prom(k)}$, (**d**) $x_{1prom(k)}$ vs. $x_{2prom(k)}$, (**e**) $x_{1prom(k)}$ vs. $x_{3prom(k)}$, and (**f**) $x_{2prom(k)}$ vs. $x_{3prom(k)}$.

In Figure 18, the phase planes of the node ND1 corresponding to the system (36) and (37) versus the representation of the average states of the six coupled nodes of the system (48) are presented, where for this case we use k=10.

### 6.3. Digital Application in Communications Using FPGA in a DSWN

We consider an experimental implementation to encrypt a signal $m_{p}\left(t\right)$ using the digital circuit with FPGAs, the signal $m_{p}\left(t\right)$ is described as follows:

(49) $$m_{p}\left(t\right)=0.042\sin\left(\frac{\pi }{180}\right)+V_{ref}$$

**Figure 19:**
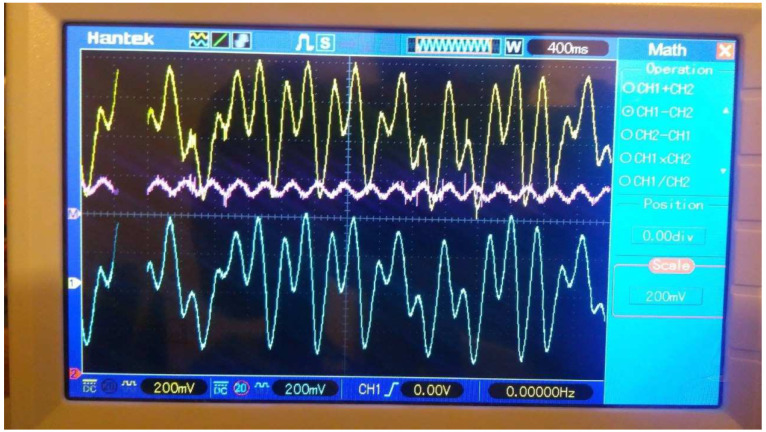
Yellow line represents chaotic carrier $x_{41}$ in the node ND4, blue line represents cryptogram $Z=x_{41}+m_{p}\left(t\right)$, and the white line represents the message recovered $\hat{m}=Z-x_{61}$ in the node ND6.

Figure 19 shows the signal of the chaotic carrier $x_{14}$ used in the node ND4 to encrypt the signal $m_{p}\left(t\right)$. The message $m_{p}\left(t\right)$ was received successfully in the node ND6.

## 7. Conclusions

We have proposed the synchronization and encrypted communication transmissions of analog and digital messages in a DSWN using Chua’s circuit as chaotic node. Analytical, numerical, and experimental studies to confirm the obtained results were conducted. We have presented a numerical method to build DSWNs starting with an iteration l=0 with N=3 nodes until iteration l=3 conformed by N=24 nodes. We have proposed the electronic implementation of DSWNs for a continuous version and also the digital implementation in a novel digital FPGA-tool Nios II embedded processor. One of the main disadvantages in the presented algorithm is that it does not have flexibility with respect to the modification of the DSWN topologies obtained at each iteration *l*, i.e., there is a fixed topology obtained at each iteration *l*. On the other hand, we believe that this work can motivate some possible future works. For example, we can build DSWNs using different chaotic nodes (or even fractional order chaotic nodes). Additionally, outwardly coupled DSWNs can be implemented in order to achieve outer synchronization. Furthermore, different components in the analog and digital implementations (OA or FPGA, respectively) can be applied, among other potential future works.

## Figures and Tables

**Figure 3:**
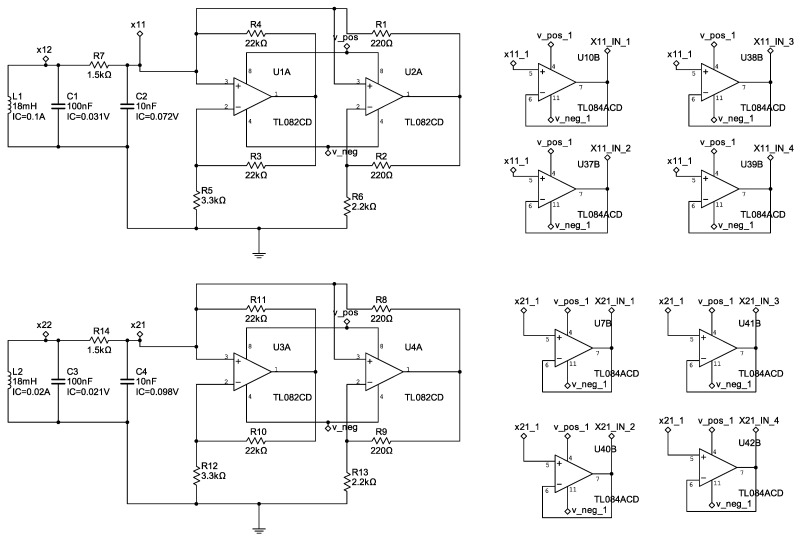
Electrical diagram of the nodes N1–N2.

**Figure 4:**
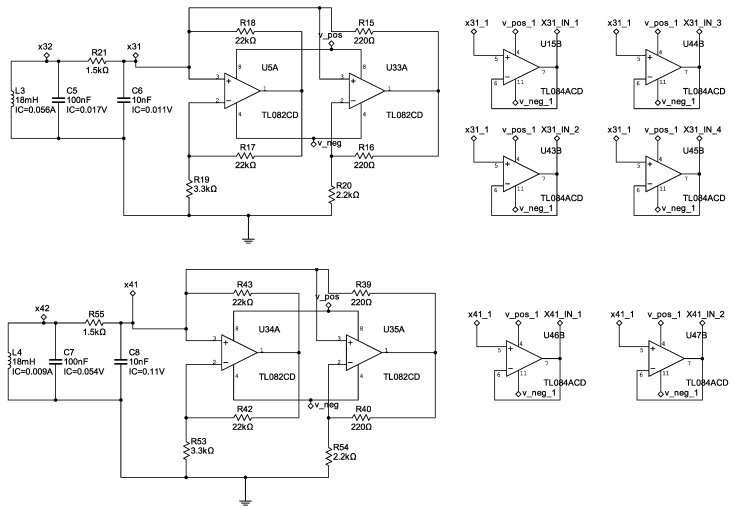
Electrical diagram of the nodes N3–N4.

**Figure 5:**
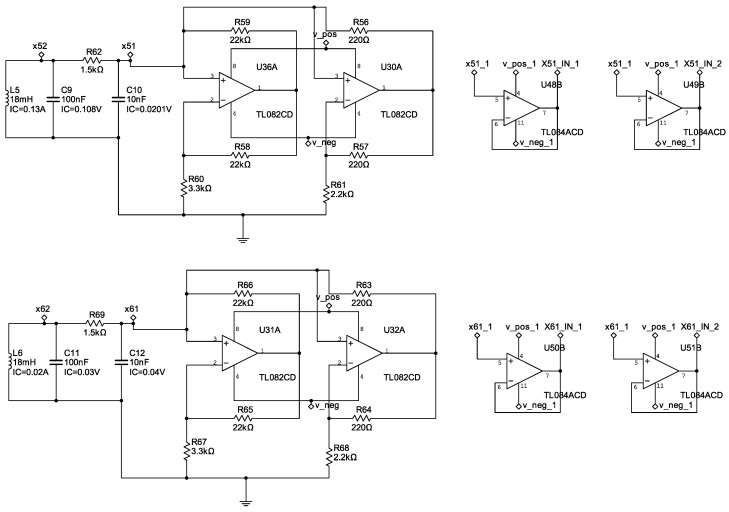
Electrical diagram of the nodes N5–N6.

## Data Availability

The data used to support the findings of this study are included within the article.
